# A Review on Development of Ceramic-Graphene Based Nanohybrid Composite Systems in Biological Applications

**DOI:** 10.3389/fchem.2021.685014

**Published:** 2021-06-29

**Authors:** Michał Jakubczak, Agnieszka M. Jastrzębska

**Affiliations:** Faculty of Materials Science and Engineering, Warsaw University of Technology, Warsaw, Poland

**Keywords:** graphene, hybrid structure, covalent modification, surface decoration, bioactivity, biosorption, composites

## Abstract

Graphene-based nanocomposites constitute an interesting and promising material for various applications. Intensive progress in the development of this group of materials offers an opportunity to create new systems useful for drinking water decontamination or other biotechnological applications. Nanohybrid structures of graphene-ceramic systems can be obtained using covalent graphene surface modification with nanoparticles (NPs) of ceramic and/or co-deposition of metals with selected morphology and chemistry. The present paper systematizes the associated bio-related knowledge and inspires future development of graphene/NPs systems. Emerging knowledge and unique research techniques are reviewed within designing the required nanocomposite structure and chemical composition, development and optimization of new methods of covalent surface modification of graphene with NPs as well as analysis of mechanisms governing the formation of covalent bonding. Further, innovative research tools and methodologies are presented regarding the adjustment of functionalities of materials used for the application in drinking water decontamination or biocidal composites. This study provides a comprehensive base for rational development of more complex, hybrid graphene-based nanomaterials with various bio-functionalities that can be further applied in industrial practice.

## Introduction

Graphene belongs both to carbon and two-dimensional (2D) materials. The term “graphene” is accepted as a monolayer of carbon atoms with sp^2^ hybridization arranged in a honeycomb crystal lattice (Novoselov et al., 2004; Neto et al., 2006). Other relevant materials include the multilayer graphene (mostly labeled as graphene, graphene flakes, or graphene platelets), graphene oxide (GO) and reduced graphene oxide (RGO). It is agreed that graphene as well as its corresponding 2D structures are named the *graphene-family nanomaterials* and labeled as GMFs (Novoselov et al., 2004).

Since the game-changing discovery of graphene, the young field of GFMs has seen a fast-growing scientific interest. The rapid increase in research on GFMs also resulted in a significant demand for further industrial use with a growing market and a significant boost in sales. The forecast market is estimated at almost 195 million USD, reaching even 1.3 billion USD by 2023, with an average annual growth rate of 47.1% (McWilliams, 2013). Consequently, the GFMs field has been concentrated on exploitation of their unique characteristics in various fields, for instance, electronics (Castro Neto et al., 2009), mechanics (Frank et al., 2007), magnetism (Ominato and Koshino, 2013), charge carrier mobility (Pallecchi et al., 2014) as well as thermal conductivity (Pop et al., 2012). Many other interesting properties were also demonstrated (Zhang et al., 2011a) and a range of possible applications of GFMs were proposed as well (Shao et al., 2010; Zhang et al., 2011a; Kucinskis et al., 2013; Petrone et al., 2013; Garg et al., 2014; Yin et al., 2014). In this regard, there are comprehensive reviews available that discuss preparation of bulk structures with high density (Markandan et al., 2017) or functional nanocomposites (Ramírez et al., 2021). They discuss the addition of GFMs as a reinforcement agent to ceramic matrices and preparation of composites as well as potential of GFMs in functional applications such as energy production and storage, piezo and thermoelectrics or electromagnetic interference shielding. It is known that presence of GFMs in ceramic matrices largely improves their functional properties, but it can be fully explored only when homogenous dispersion of GFMs into ceramic matrix is provided. On the other side, preparation of GFMs surface-grafted and functionalized with a thin layer of ceramic material is not fully explored due to lack of complete understanding of mechanisms governing ceramic layer formation. Application of such structures in biological field is therefore hindered, due to lack of rational designing and synthesis of GFM-based ceramic.

**FIGURE 1:**
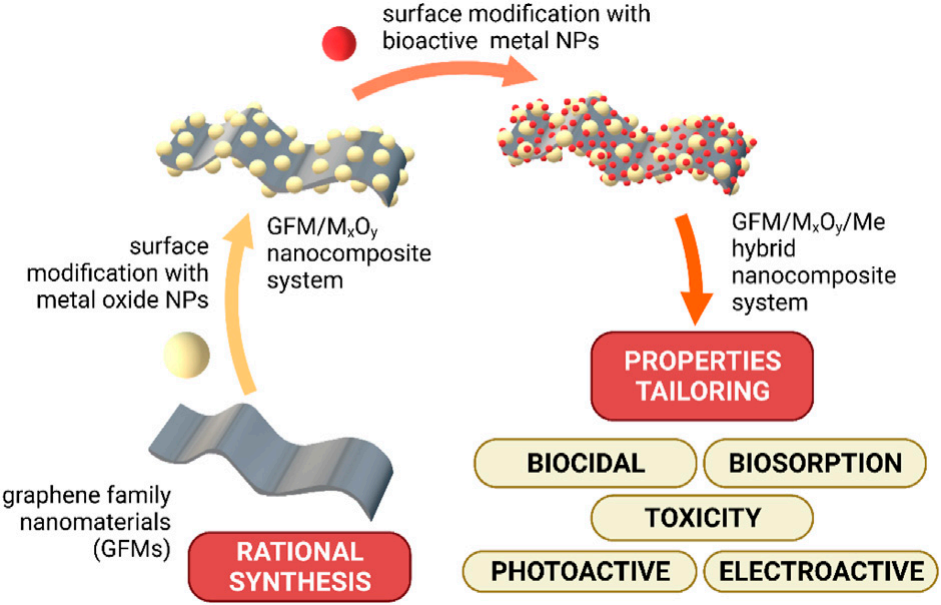
Schematic of the areas of research covered by this work.

Given the abovementioned knowledge gaps, this review papers summarizes and discusses the emerging knowledge and unique research techniques used toward development of graphene-based nanocomposites. In this regard, modification with ceramic nanoparticles (Al_2_O_3_ TiO_2_) and bioactive metals (noble metals etc.) appeared recently to be the most interesting in terms of e.g., bioactivity. Designing the required nanocomposite structure and chemical composition is highlighted together with development and optimization of new methods of GFMs covalent surface modification with NPs as well as analysis of mechanisms governing the formation of covalent bonding. Further, innovative research tools and methodologies are presented regarding the adjustment of material functionalities (biocidal, biosorption, toxicity, photoactive and electroactive) used for the application in drinking water decontamination or biocidal composites. These were summarized in Figure 1.

## Bioactivity and Toxicity of GFMs

**FIGURE 2:**
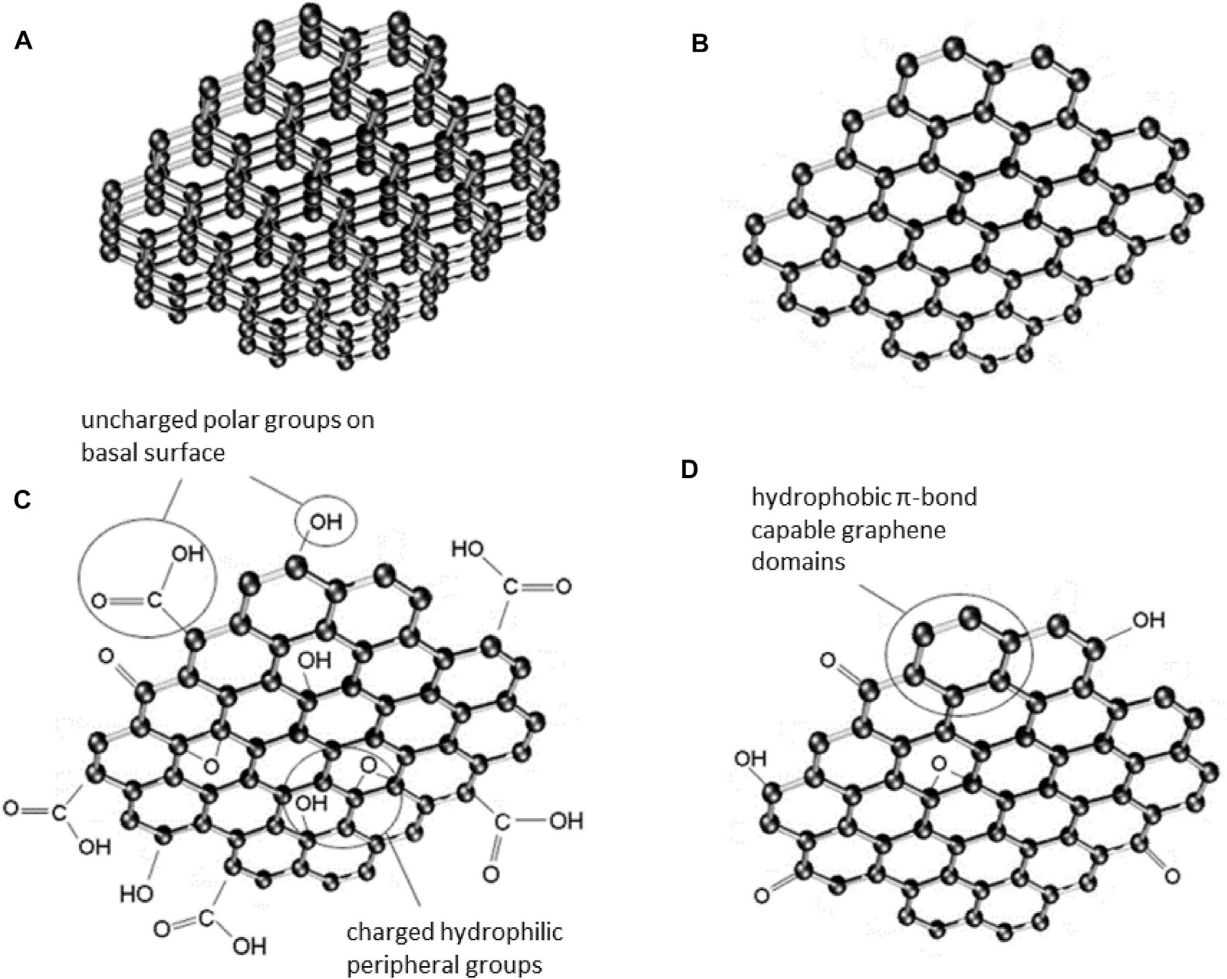
Schematic of the graphene-family nanomaterials (GFMs) structures such as multilayered graphene flake **(A)**, graphene sheet **(B)**, graphene oxide (GO) **(C)** and reduced graphene oxide (RGO) **(D)**. For simplicity, the structures **(C**,**D)** are presented in a form of carbon monolayers (Reproduced with permission from (Jastrzębska et al., 2012)).

**FIGURE 3:**
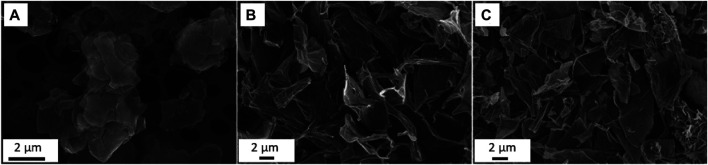
Comparison of morphologies of graphene flakes **(A)**, GO **(B)** and RGO **(C)** using SEM analysis (Reproduced with permission from (Jastrzȩbska et al., 2017a)).

It is accepted that the hydrophobic nature of graphene limits its potential application (Wang et al., 2013). Such limitations however may be overcome due to the chemical oxidation of graphene, when the oxide form of graphene is formed. Highly oxidized GO is filled with lots of edges-located and oxygen-containing functional groups such as carboxyl, hydroxyl and epoxy (Song et al., 2013) (*see* Figure 2). On the other hand, reduced graphene oxide obtained by thermal and chemical reduction methods, contains much less oxygen-based functional groups. The presence of these groups, their number or absence determines the properties of GMFs, and therefore their functionality (Song et al., 2013; Olborska et al., 2020). Figure 3 shows Scanning Electron Microscopy (SEM) images of morphologies of the studied GFMs. GFMs differ not only in the case of surface chemistry but also in structural properties such as morphology, size, and shape of the flakes as well as their thickness. Despite having similar chemical structures, both single and multilayer graphene possesses different morphologies. As clearly shown in work (Sarikaya et al., 2020), multilayer graphene has a very wrinkled, cotton flower-like morphology, while the surface of single flakes of graphene is basically flat. Differences were noticed not only in the case of morphology, but also dimensions, as for multilayer graphene the lateral size was about 5 nm, while for single flakes it was less than half of this value. In work (Gomez et al., 2020) authors also observed wrinkled structure for graphene oxide and reduced graphene oxide with the thickness in the range of 1 nm. The lateral size however was substantially bigger, and as they found out, it was about 20–30 μm for both GO and RGO. As further studies revealed, GO has a tendency to retain smooth, slightly wrinkled surface, while RGO sheets stacks and aggregates with the creation of rough and pointed edges were observed (Gurunathan et al., 2012).

**FIGURE 4:**
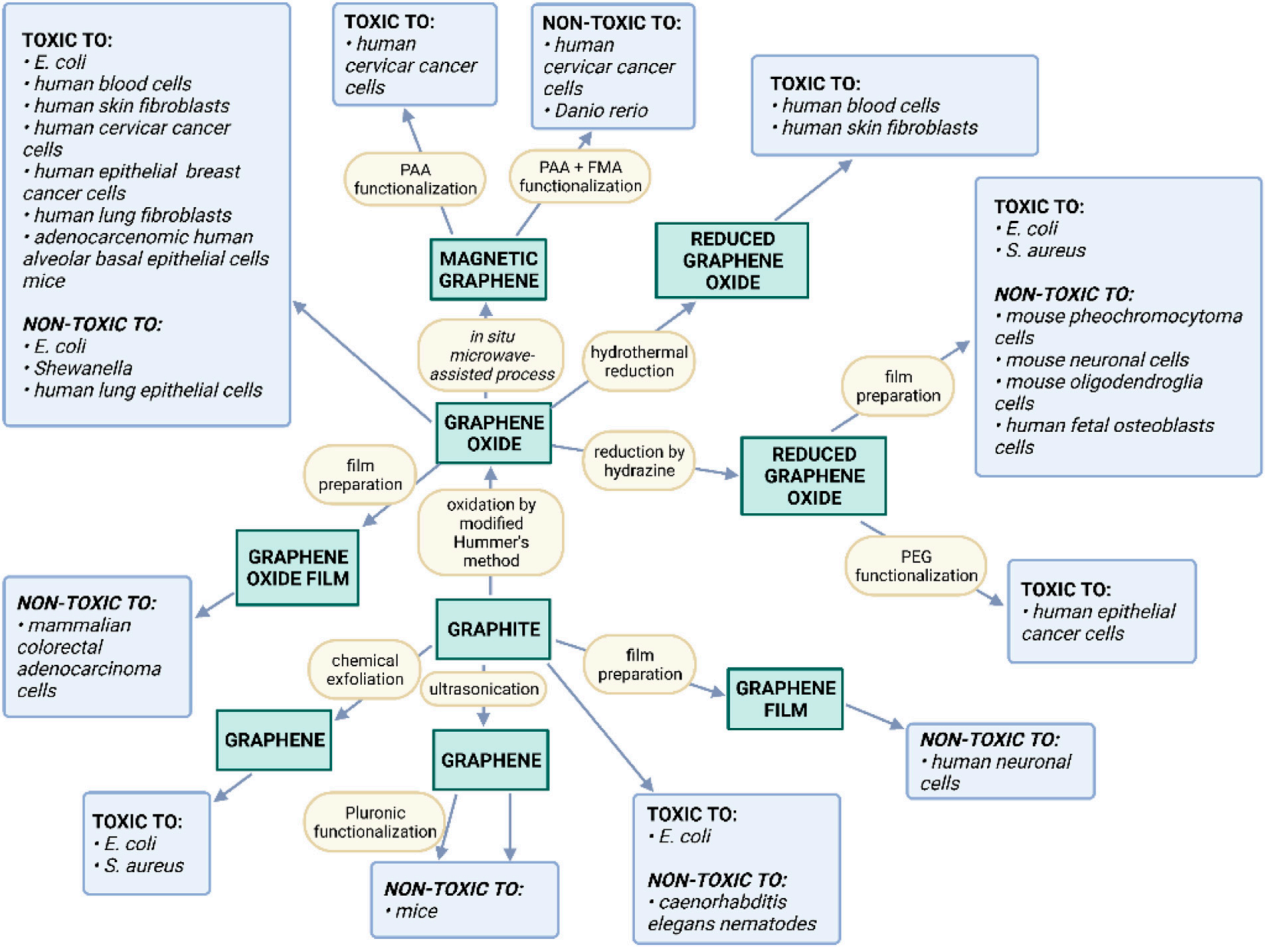
Summary of the observed toxic effects of GFMs in terms of their production methods and the impact of possible functionalization (Reproduced with permission from (Jastrzębska et al., 2012)).

Recent studies also suggest the interesting biological properties of GFMs. For instance, it was demonstrated that GO and graphene flakes interact with many biomolecules. Consequently, they may pose an impact on various biological systems (Novoselov et al., 2004; Radic et al., 2013). The rapidly growing volume of literature on the subject shows that the development of GFMs-based bioactive nanocomposite systems shows both opportunities and challenges (Li et al., 2008). The important aspect to analyze is the potential toxicity of GFMs towards various living organisms and the relevant impact of surface modifications. Figure 4 shows a constellation of the observed toxic effects of GFMs in terms of their production methods. There is no doubt that the bioactivity of individual GFMs may greatly vary. Nevertheless, it is closely associated with the GFMs production method and their possible surface modification. This in turn determines both their morphology and physicochemical properties.

### Biocidal Characteristics of GFMs

Antibacterial properties of different representatives of GFMs also suggest their application potential in biocidal products (Akhavan and Ghaderi, 2010; Hu et al., 2010). While considering their application in water filtration systems, the most significant is their interaction with bacterial cells (Akhavan and Ghaderi, 2010; Hu et al., 2010; Zhang et al., 2011a). The most popular GFMs studied in this context are multilayer graphene flakes, GO and RGO. It is demonstrated that RGO exhibits higher antimicrobial activity towards the *Escherichia coli* and *Staphylococcus aureus* strains in comparison to GO (Hu et al., 2010). The assumed toxicity mechanism is the result of direct contact of cells with GFM, whereby *E. coli* shows more resistance compared to *S. aureus*. Other results suggest the antimicrobial activity of GO against *E. coli* and *S. aureus* and a mechanism of toxicity stemming from the contact damage to the cell wall (Hu et al., 2010). Further studies indicate stronger toxic effects of GO than RGO towards *E. coli* and the oxidative stress is mostly indicated as an additional toxicity mechanism (Liu et al., 2011). Apart from these, three other studies showed a lack of bactericidal effect of graphene flakes (Zhang et al., 2011a) and GO (Wang et al., 2011; Akhavan and Ghaderi, 2012) towards bacterial cells. There was no reduction of bacterial growth in presence of selected GFMs (Akhavan and Ghaderi, 2010; Hu et al., 2010; Liu et al., 2011). Therefore, a follow-up study thoroughly analyzed these issues and showed a significant impact of the methods of production of individual GFMs on their toxic characteristics (also against bacteria) (Jastrzębska et al., 2012).

Given the abovementioned information, GO is the most interesting 2D nanomaterial in terms of potential bioactivity, including both biocidal properties (Akhavan and Ghaderi, 2010; Hu et al., 2010; Kurantowicz et al., 2015) and biocompatibility (non-biocidal features) (Zhang et al., 2011a; Wang et al., 2011). Therefore, three representatives of GFMs such as graphene flake, GO and RGO were compared in (Jastrzȩbska et al., 2017b). At a first glance, it becomes clear that the morphology of graphene flakes and GO together with RGO may play a key role in occurring the bio-nano interactions. However, obtained results indicate other important factors governing the toxicity towards bacteria. Apart from the morphology, these are mostly a type of bacteria cells. Indeed, the absence of bactericidal activity of GO was demonstrated in the presence of *E. coli, S. aureus, Bacillus sp.* and *Sarcina* (Jastrzȩbska et al., 2017b), which is consistent with other works (Zhang et al., 2011a; Wang et al., 2011).

### Biosorption Properties of GFMs

Another interesting biological research approach assumes that the produced GFMs could be applicable in drinking water filtration and therefore, should exhibit both biocidal and biosorption properties towards bacteria cells. The best choice of GFM should be based on not only antibacterial effects, but also the biosorption properties. Many carbon-based nano-sorbents are widely used in water decontamination from various undesirable inorganic and organic substances. Compared to their macroscopic counterparts, the advantage of nano-sorbents in this field is a significantly larger specific surface area. The effective adsorption properties were already proved for nonmodified carbon nanotubes (Li et al., 2003; Li et al., 2004), carbon nanotubes surface-modified with inorganic NPs (Peng et al., 2005), carbon fibers (Mangun et al., 2001) as well as nanoporous ceramics (Yantasee et al., 2003).

A recent study (Jastrzȩbska et al., 2017b) compared the biosorption characteristics of graphene flakes, GO and RGO. It also confirmed the applicability of zeta (ζ) potential in the experimental analysis of GFMs’ biosorption properties. It is accepted that the electrostatic charge present on the surface of various materials (determined experimentally as ζ) is the key factor for attracting bacteria towards the surface (Marshall et al., 1971; Bitton and Marshall, 1980; Mozes et al., 1987; Martinez-Martinez et al., 1991). To date, extensive research has been conducted on the occurrence of the electric double layer and its linking directly to the zeta potential and the subsequent adsorption of bacteria cells onto the solid surface (Fletcher and Loeb, 1979; Ellwood et al., 1982; Scholl et al., 1990; Gannon et al., 1991; Krekeler et al., 1991; Marshall, 1992; Huysman and Verstraete, 1993). It is noted that these studies concerned, to a significant extent, the interaction between the surface of inorganic material and the bacteria and pointed to the necessity of an individual approach towards understanding this phenomenon due to differences in the chemical composition of materials’ surface (Fletcher and Loeb, 1979; Ellwood et al., 1982; Scholl et al., 1990; Gannon et al., 1991; Krekeler et al., 1991; Marshall, 1992; Huysman and Verstraete, 1993). Yet, there is no doubt that the phenomenon of biosorption is still difficult to describe and to examine experimentally since the structure of and surface features of living organisms are much more varied and complex than the surface of inorganic materials (Kłodzińska et al., 2010). For instance, it was reported that the electrostatic charge that forms on the cell wall surface may be the result of dissociation of inorganic functional groups such as carboxylic or amino acid exposed to the aquatic environment (Dikusar, 1940; Santoro and Stotzky, 1968; van Loosdrecht et al., 1987).

What is more, the surface of GO hydrophilic is highly negative ζ which allows for the preparation of stable water dispersions (Li et al., 2008). It is explained by the presence of electrostatic repulsion which allows the preparation of stable water dispersions of GO. Consequently, it becomes clear that the material-to-cell interactions cannot be governed only by electrostatic interactions. However, the very first studies did not concern the processes of biosorption. A later study (Kurantowicz et al., 2015) demonstrated the changes in the ζ of GFMs due to their interaction with bacteria. Thus, taking into account the state of knowledge on biosorption, it was important to carry out a thorough investigation of the biosorption efficiency of GFMs to different bacteria using ζ parameter. It was demonstrated that the biosorption efficiency of each GFM toward *E. coli, S. aureus, Bacillus sp.* and *Sarcina* species is different and depends on the type of strain. The zeta potential of *E. coli* and *S. aureus* strains after their adsorption on the GFM surface did not undergo many changes (Jastrzȩbska et al., 2017b). In general, carbon materials are considered to be the primary food source for bacteria (carbon source) (Fonte et al., 2013). While following the studies (Soni et al., 2008) and (Bruinsma et al., 2001), it is assumed that bacteria should not change their ζ as a result of adsorption, but retain their natural (healthy) ζ value, determining their good viability. Finally, this assumption was confirmed only for *E. coli* and *S. aureus* strains. Different results were obtained for the *Bacillus sp.* strain, for which ζ changed its value from −26 mV to −5 mV due to the adsorption, compared to the pure cell suspension. Such a result indicates a lack of adsorption and transformation of *Bacillus sp.* cells into the state of nutrition deficiency following the assumptions made in other studies (Bruinsma et al., 2001; Soni et al., 2008).

Another study considered GO flakes using Fourier transform infrared (FTIR) and Raman investigations (Jastrzębska and Olszyna, 2015). It confirmed that the GO’ surface functional groups (i.e., C=O, O=C-OH or C-OH) take part in amphoteric reactions resulting in the formation of surface charge and zeta potential. It also showed a strong association of -COOH and -OH groups leading to the formation of higher-level (e.g., ionic doublets, triplets), neutral or charged associated molecules. The obtained results excluded the participation of C=O groups in amphoteric reactions, contrary to -COOH and -OH groups.

**FIGURE 5:**
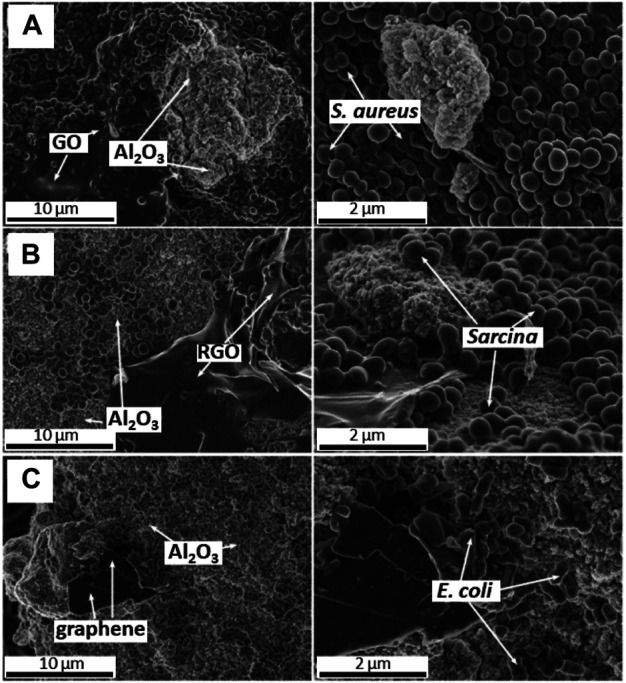
SEM images of competitive biosorption between nano-Al_2_O_3_ and GO towards *S. aureus* cells **(A)**, RGO towards *Sarcina* cells **(B)** and graphene flakes towards *E. coli* cells **(C)** (Reproduced with permission from (Jastrzębska et al., 2017b) with minor changes).

Further comparison of biosorption characteristics of the GFMs considered the presence of aluminum nano-oxide (nano-Al_2_O_3_) as a competitive factor (Jastrzȩbska et al., 2017b). It was found that the nano-Al_2_O_3_ exhibited much stronger biosorption properties in comparison to GO and often better than other investigated GFMs. The SEM analysis allowed observing the majority of bacterial cells (e.g., the *S. aureus* strain) preferred to colonize the GO surface in the presence of nano-Al_2_O_3_ (Figure 5A). Another case showed *Sarcina* cells preferring the colonize of nano-Al_2_O_3_ in contrary to RGO (Figure 5B). Also, *E. coli* tended to colonize mainly the surface of nano-Al_2_O_3_ while graphene flakes were present in the suspension (Figure 5C).

## On the RGO/Ai_2_O_3_ Nanocomposite System

### Synthesis Approaches

The production of nanocomposite materials *via* modification of GFMs surface with NPs is an interesting research topic. Such an approach can be applied to developing innovative water filtration technologies. The GFMs representative that appears as the most interesting in this regard is the GO due to its stronger biosorption characteristics as compared to graphene or RGO. The relevant literature points to the possibility of obtaining graphene nanocomposites using various methods of surface decoration, whereby methods that allow for the covalent attachment of NPs are the most perspective for avoiding NPs’ leakage. This advantage resulted in large interest in the covalent modification of GFMs surface with ceramics nanoparticles (Zhang et al., 2010; Shen et al., 2010; Wu et al., 2010). Obtaining a non-agglomerated well-defined core-shell structure of 2D nanocomposite flakes is a great challenge because it requires the development of techniques for controlling material morphology and structure. Yet, the first attempt to obtain graphene/NPs in a form of single 2D flakes surface-modified with a thin covalent NPs layer has considered pure graphene flakes, but without a breakthrough. Importantly, the aim is to obtain an adequate and strong bonding between the GFM surface and NPs with a sustainable approach. Meanwhile, it was observed that GO offers the greatest possibilities of surface modification thanks to the presence of oxygen-containing surface functional groups. In this regard, the GO was soon accepted as the most promising material for such a purpose. Consequently, surface-modifications of GO using wet (involving water) and sol-gel methods became highly explored. For instance, RGO/SiO_2_ nanocomposite with a thin SiO_2_ layer was obtained for use as transparent conductive tracers (Watcharotone et al., 2007). Another study presented a solid RGO/Al_2_O_3_ nanocomposite for catalytic hydrodesulfurization (Jiwei et al., 1987). The synthesis method consisted of wet hydrolysis of boehmite in an acidic environment, GO modification, and subsequent heat treatment.

Based on previous results, it was found that Al_2_O_3_ nanoparticles should be primarily used as the modifier of bioactive properties of GFMs (Jastrzębska et al., 2017b). The objective was to obtain a material that would combine the beneficial bioactive properties of both GO and nano-Al_2_O_3_. The most important achievement in this field is the development of a novel *dry* (without using water) sol-gel method for obtaining a highly demanding system of graphene covalently modified with aluminum oxide nanoparticles (Jastrzębska et al., 2015). The method involves a reactive metal-organic aluminum compound (triethylaluminium) as the reagent and source of Al in a water and oxygen-free environment (Jastrzębska et al., 2015). The analysis of the final powdered product confirmed that Al_2_O_3_ nanoparticles covered uniformly the RGO surface, while the GO was reduced to RGO (Jastrzębska et al., 2016a). During the process, triethylaluminium reacts with terminal functional groups (i.e., C=O, O=C-OH, C_2_O, C-OH) present on the surface of GO. As a result, oxygen is being transferred from the GO surface to the structure of the emerging alumoxane precursor. This effect is called the *in situ* reduction of GO to RGO. In the next stage, organic precursor groups are involved in the formation Al_2_O_3_ crystal structure. As a result, the RGO/Al_2_O_3_ nanocomposite is forming *via* the evaporation of hexane and precursor thermal decomposition.

**FIGURE 6:**
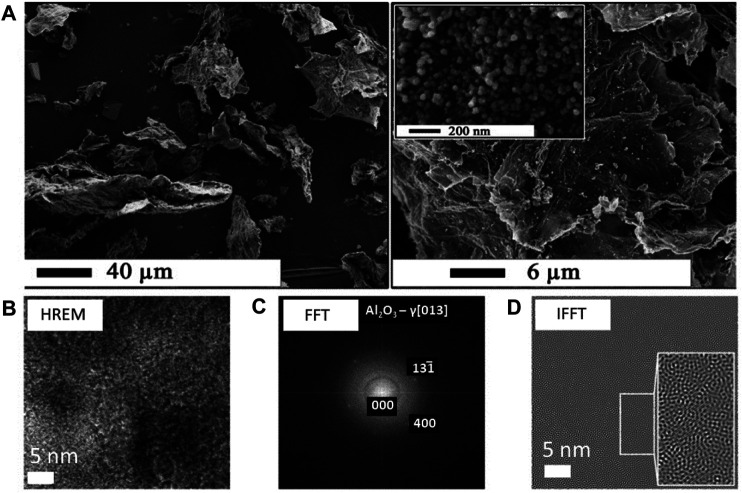
SEM and TEM analysis of the RGO/Al_2_O_3_ nanocomposite morphology **(A)** and structure **(B**–**D)** obtained *via* simplified sol-gel method (Reproduced with permission from (Jastrzębska et al., 2016a) with minor changes).

On the other hand, the new *dry* sol-gel method for RGO/Al_2_O_3_ nanocomposite synthesis turned out to be troublesome because of the high reactivity of the Al_2_O_3_ precursor and the need for an inert environment of the process. The inert environment requires the use of complicated equipment for operation in an argon protective atmosphere. Also, the potential risk of explosion or spontaneous reactant ignition was assessed as problematic from the upscaling point of view. Thus, it soon became necessary to develop a safer as well as more sustainable, simplified, and user-friendly method to produce the RGO/metal oxide nanocomposites. The modification of RGO with Al_2_O_3_ nanoparticles using less reactive and safer reagents constitutes yet another significant contribution to the development of the RGO/Al_2_O_3_ nanocomposites (Jastrzębska et al., 2016a). The newly developed process of covalent GO surface modification with Al_2_O_3_ nanoparticles was called the *simplified sol-gel method* (Jastrzębska et al., 2016a). It utilizes an alumoxane Al(OR)_3_ compound (R is an alkyl substituent) as the precursor for Al_2_O_3_. What’s important, in case of the application of aluminium alkoxy compounds as reagents, alcohols can be used as solvents. The new method of RGO/Al_2_O_3_ nanocomposites synthesis allowed to obtaining similar results of material morphology and properties, as compared to a previous study (Jastrzębska et al., 2015). The FTIR and Raman analysis of the intermediate product (precursor) before thermal decomposition confirmed the presence of covalent bonding between Al and O atoms at the interface between GO surface and alumoxane. In particular, the C=O (1730 cm^−1^), C-O-C (1280 cm^−1^) and C-O (1225 cm^−1^) groups formed a covalent Al-O bonding (Jastrzębska et al., 2016a). The intensity of the FTIR signals corresponding to these groups was significantly decreased, while the signals corresponding to the Al-O bonds appeared in the FTIR spectra. The Raman analysis showed no changes in the hexagonal structure of graphene within mutual intensities of “D” and “G” signals, characteristic for GO. While using the thermogravimetric analysis (TGA) combined with FTIR, a detailed analysis of the process of thermal decomposition of the precursor into the final product (the RGO/Al_2_O_3_ nanocomposite, *see* Figure 6) was carried out. On this basis, the optimum temperature of thermal decomposition was determined (at 300°C) (Jastrzębska et al., 2016a), which allowed for conducting the complete process of thermal decomposition (production of the RGO/Al_2_O_3_ nanocomposite) while maintaining the graphene structure (no hexagonal structure degradation). The further X-ray photoelectron spectroscopy (XPS) analysis also confirmed the occurrence of *in situ* GO reduction to RGO. The Al_2_O_3_ nanoparticles were, therefore, undoubtedly attached to RGO *via* a covalent bonding.

Further studies concerned the optimization process of nanocomposites prior to upscaling. It was most important to find the lowest possible agglomeration of RGO/Al_2_O_3_ nanocomposite flakes and the most homogeneous coverage of the RGO surface. It was demonstrated that the synthesis parameters have a decisive influence on the morphology and physicochemical properties of the RGO/Al_2_O_3_ nanocomposites (Jastrzȩbska et al., 2017b). An important result is that too intensive heat treatment leads to the agglomeration of Al_2_O_3_ nanoparticles on the edges of RGO flakes. It should be noted, however, that while this phenomenon cannot be completely eliminated, it can be controlled by the appropriate selection of both synthesis and heat treatment parameters of the intermediate product. The presented results confirmed that despite the occurrence of less wettability (Wang et al., 2008), RGO may be also applicable for surface modification with ceramic oxides due to the presence of the residual quantities of functional groups on the surface.

Other works concerned a newly developed solvothermal method, which was employed for the first time to fabricate RGO/Al_2_O_3_ nanocomposite in a form of Al_2_O_3_ nanorods covering the GO surface (Ikram et al., 2018). Further reduction of GO to RGO was obtained by further calcination treatment, which was followed by hot pressing to obtain hybrid samples. It has been noted that different concentrations of RGO could be obtained with varying times of calcination, however, the enhanced crystallinity was achieved by increasing calcination temperature. The solid RGO/Al_2_O_3_ samples were characterized by enhanced electrical, mechanical, thermal, and physical properties and therefore both calcination and hot pressing are beneficial for the formation of Al_2_O_3_ nanostructures and GO/RGO transformation. The enhanced mechanical properties of RGO/Al_2_O_3_ were associated with covalent-type chemical interactions and efficient mass diffusion between RGO and Al_2_O_3_ in the grain boundary regions, while improved physical properties were attributed to the well-aligned, elongated and fine nanorod morphology of Al_2_O_3_. The authors also observed enhancement of the electrical conductivity due to the more availability of surface electrons coming from RGO. The restoration of the sp^2^ C-C bonds and cross-linking between RGO sheets additionally caused the improvement of electrical properties. It is also noted that the heat treatment was crucial in both cases.

While considering the incorporation of RGO to the alumina matrix, the important aspect to consider is controlling the growth of the Al_2_O_3_ grain by the presence of graphene sheets. The Al_2_O_3_ grains size was remarkably reduced due to the presence of graphene sheets (He et al., 2009). It was observed, however, that the presence of alumina particles enhances the milling efficiency, which was used to prepare investigated samples. It was also shown that the 0.5 wt% addition of GO to Al_2_O_3_ matrix *via* powder metallurgy and spark plasma consolidation (SPS) allow for a significant increase (almost 35%) of the fracture toughness for GO/Al_2_O_3_ composites in comparison to pure alumina sinters (Cygan et al., 2017). Also, in this case, a good interface between the reinforcement and the matrix was revealed using TEM. Therefore, the expected outcome is a significant improvement of fracture toughness (60%) for the RGO/Al_2_O_3_ composites (Wozniak et al., 2017). Mechanical milling, as well as solution-based synthesis, were combined and employed in recent work (Kim et al., 2018) to achieve RGO/Al nanocomposites in the so called mechano-chemical process. In the first step, Al grains are milled into flakes with the increased surface area. Then, they are coated with GO flakes *via* a solution process with the assistance of polyvinyl alcohol for the formation of hydroxyl groups. In the last step, the composite powder is subjected to drying, annealing, milling, and consolidation. The large surface area is provided by mechanical milling and an overall improved dispersion of RGO. This adjusts the mechanical and thermal properties of the nanocomposite.

In recent years, bio-inspired nanocomposites have caught scientists’ attention due to their sustainability and robustness. In work (Chen et al., 2021), the monolithic RGO/Al_2_O_3_ nanocomposites with fibrous bamboo-like architecture were fabricated for improved tribological performance and wear mechanisms. The materials were obtained by a coating of Al_2_O_3_ fiber cells with GO *via* physical adsorption. The preforms were then rearranged by the molding process. The last step involved removing the organic binder in a high vacuum and subsequent hot-pressing to obtain the final structure. The presence of RGO boundaries between Al_2_O_3_ fiber cells resulted in superior friction reduction and wear resistance, with stable friction coefficients. Fracture responses of bioinspired RGO/Al_2_O_3_ fibrous monolithic ceramic were also investigated in work (Chen et al., 2020a). The investigated nanocomposite was characterized by progressive plastic failure behavior, excellent damage tolerance and high structural reliability due to complex hierarchical architectures with several different levels. The obtained structure enabled crack deflection, delamination and redistribution of load, which enhanced fracture resistant behavior.

### Potential Environmental Implications

**FIGURE 7:**
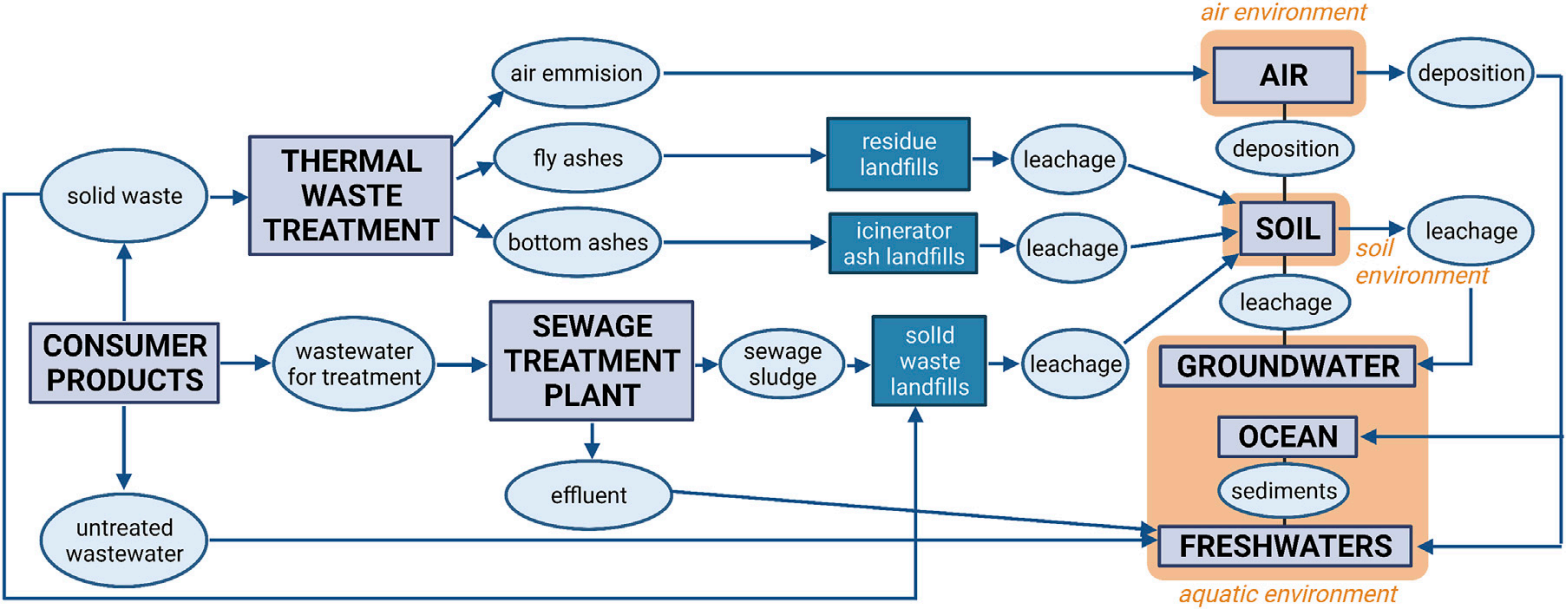
Schematic of potential transfer pathways of the RGO/Al_2_O_3_ nanocomposite in the natural environment as a result of their release from consumer products. Arrows correspond to the directions of transfer between the environmental biota (Reproduced with permission from (Jastrzębska and Olszyna, 2015) with minor changes).

**FIGURE 8:**
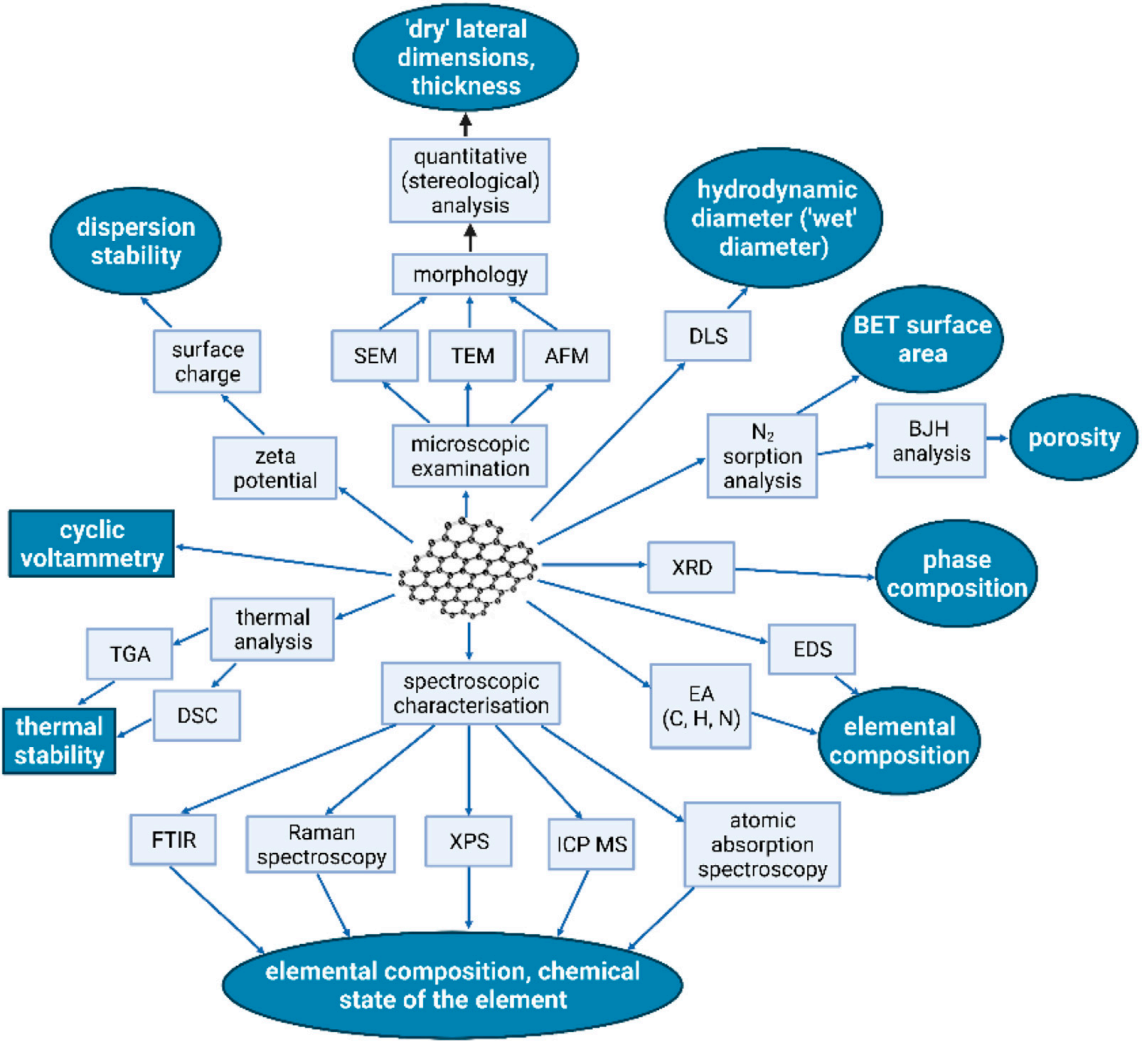
Most important methods and research techniques applied in the characterization of graphene-family nanomaterials (GMFs). Fields marked in grey color represent GFMs parameters directly linked to their bioactive properties. For simplification, the following abbreviations were used: TEM, transmission electron microscopy; SEM, scanning electron microscopy; AFM, atomic force microscopy; DLS, dynamic light scattering; FT-IR, Fourier-transform infrared spectroscopy; XPS, X-ray photoelectron spectroscopy; XRD, X-ray diffraction; BET, Brunauer–Emmett–Teller; BJH, Barrett Joyner, Halenda; EDS, Energy-dispersive X-ray spectroscopy; TGA, Thermogravimetric analysis; DSC, differential scanning calorimetry; ICP-MS, inductively coupled plasma mass spectrometry (Reproduced with permission from (Jastrzębska and Olszyna, 2015) with minor changes).

Simultaneously with research on the applicability of graphene nanocomposites in the processes of biosorption, the study (Jastrzębska and Olszyna, 2015) explores the threats arising from the possibility of leaking of the developed nanocomposites in purified water and the relevant impact after further spreading into the environment. At this stage, it was necessary to determine the potential risks associated with the production of nanocomposites on an industrial scale and their use in consumer products. An essential outcome of the study (Jastrzębska and Olszyna, 2015) is the analysis of the probable distribution and transport paths of graphene nanocomposites in the environment and the associated threats for particular ecosystems. The analysis carried out on exemplary RGO/Al_2_O_3_ nanocomposite incorporated elements of the life cycle assessment (LCA) concerning threats associated with the use of the RGO/Al_2_O_3_ in drinking water filtration. Figure 7 presents a diagram that analyses the potential transport pathways of the developed RGO/Al_2_O_3_ nanocomposites into the natural environment. The assumed case-study scenario involved the environmental fate of the RGO/Al_2_O_3_ after its leakage from a manufacturing plant (filtration material production), transportation to another plant, storage, leakage from filtration material, dusting, and storage of the used filter as solid waste. In the case of threats associated with leaching, it was observed that the sludge can be used for agricultural soils, disposed of as solid waste in landfills, or burned. The potential risk also depends on whether the RGO/Al_2_O_3_ interacts with the various environmental components such as soil, sediments, fresh and groundwater resources, wastewater and marine environment. The analysis pointed out that the exposure paths depend not only on the fate and transfer pathways of the RGO/Al_2_O_3_ in the environment but also on the factors affecting its transformation (aggregation, limiting factors, environmental conditions). In this regard, Figure 8 additionally shows the schematic summary of the most important methods and techniques that can be used as the so called *selection key*s for the characterization of GFMs in terms of targeted research on their bioactive properties and potential toxicity.

In view of the potential bioactive properties of GFMs, special attention should be given to the selection of the dispersion media (Jastrzębska and Olszyna, 2015). It is concluded that such properties should be examined in conditions as close to the natural environment as possible, which is currently being applied in the research on other types of nanomaterials (Keller et al., 2010). The issue of the significance of the zeta potential analysis is also a matter of interest (Jastrzębska and Olszyna, 2015). The theory of the electric double layer may be successfully used in the case of graphene oxide (Li et al., 2008) mainly on account of the hydrophilic properties of its surface. Thanks to this, GO flakes can form a stable colloidal dispersion, which contrasts with graphene and reduced graphene oxide that possess water-repellent surfaces (Stankovich et al., 2006; Stankovich et al., 2007). It is necessary to check the ζ of RGO/metal oxide suspensions to enable better anticipation of the processes of aggregation and deposition that are responsible for further material transfer in the environment. Following the same logic, the study (Jastrzębska et al., 2016a) examined ζ of the developed GO/Al_2_O_3_ (40 %wt.) nanocomposite flakes. The reference material was GO flakes and pure Al_2_O_3_ nanoparticles (not attached to RGO surface). The study was performed in a wide pH range in distilled water, electrolyte solution (NaCl) and drinking water environment. Specific attention was paid to the analysis of the shape of ζ curves. Obtained results have shown a strong dependence of ζ value on the pH level and the chemical composition of water. These were, in turn, closely linked to changes in the chemical composition of materials’ surface (Mikolajczyk et al., 2015). The interesting observation is the mitigating effect of the RGO core on measured ζ values for the developed GO/Al_2_O_3_ nanocomposite system. Such changes can influence the environmental risk of GFMs.

In general, it is presumed that graphene does not possess a risk to the environment due to the small amounts produced and used as well as its uncomplicated structure, consisting only of carbon. It may, however, hold a threat, especially under certain conditions and in different environments (Arvidsson et al., 2018). Similar ecotoxicological effects can be assumed for other GFMs. However, some possible risks were noticed for GFMs in regard to their potential toxicity (Arvidsson et al., 2011; Jahnel, 2015). As there is a lack of studies considering RGO/Al_2_O_3_ ecotoxicity, it is possible to consider RGO and Al_2_O_3_ components separately.

The ecotoxicity of GFMs has been presented in detail in a recent work (Jastrzębska and Olszyna, 2015). Other studies have shown that toxicity of RGO/NPs synthesized *via* chemical reduction of NPs precursor depends on the surface properties of investigated organisms as well as chosen nanomaterials (Yin et al., 2020). The more hydrophobic and complex cells of *C. reinhardtii* were more sensitive than *S. obliquus*. Differences in the properties and chemistry of algal cells were related with increased toxicity, due to the more intense metal ion adsorption and interactions with NPs. What is more, induction of cellular oxidative stress, as well as membrane damage, occurred more often in case of RGO/NPs nanocomposites with more complex heterointerfaces. The synthesis method and properties of nanocomposites were only partially responsible for the toxicity, while the algal surface property was the main factor. Cellular metal accumulation and membrane damage due to the interactions with metallic NPs is disturbed, because of the presence of defensive mechanisms like metal pumping and secretion of exudates, as well as complex and heterogeneous composition of algae surface, which support binding of metals.

What is more, the hydrophobic surface of microalgae have a greater potential for biological interactions, which then disturb interconnections with hydrophobic nanomaterials. Further, in case of *C. reinhardtii*, monocarboxylic acids and amide groups, as well as polypeptides in outer surface of cell wall, which play a dominant role during the process of metal ion binding were observed. The short-term exposition of an ocular region in mice to RGO (synthesized *via* a hydrazine reduction) showed a much lower risk than in case of GO (obtained according to Hummers’ method). A short-term exposure to GO caused apoptosis, necrosis, cell cycle alteration, and eyeball cell death (An et al., 2018). Contrary, both commercially available GO and chemically reduced RGO were not highly toxic to mussel hemocytes (Katsumiti et al., 2017). These GFMs can be, however, internalized into the cells, therefore increasing ROS formation, mitochondrial and lysosomal dysfunction, and cell death. It was concluded, that toxicity of GFMs may be also influenced by physicochemical properties (e.g., lateral dimensions), as well as increased stability, corresponding to higher availability and bioaccumulation inside the cells. The toxicity of different concentrations of GFMs to zebrafish embryos was investigated at the early stage of fish development (Liu et al., 2014). In case of RGO, synthesized by using a hydrazine GO reduction process, mortality, as well as significant development abnormalities (e.g., tail detachment, somite formation), was not detected. However, it was found that RGO affects significantly the hatching rate. The larvae length has been also affected to some extent, which can be related to the interference with hatching enzyme and hypoxia in zebrafish, due to the oxygen exchange disturbance.

Recent work (Załęska-Radziwiłł et al., 2020) considered the effect of aluminum oxide nanoparticles on aquatic organisms. It was revealed that long-term exposure (28 days) of microcosm caused the reduction of biodiversity of microbenthic and plankton organisms, with increased activity of antioxidative enzymes of benthos. It was also observed that nanoparticles tended to accumulate on the surface of *Daphnia magna* and in its mitochondria. This was not, however, the case for the bulk counterparts of nanoparticles. The toxicity of nano and bulk Al_2_O_3_ towards *Caenorhabditis elegans* was compared in work (Wang et al., 2009a). The authors observed significant differences in toxicity of nano, micro and macroparticles, as shown by the lethality of the investigated worms, the number of eggs inside the body, and offspring per nematode.

The abovementioned results are of particular importance because they suggest that if the RGO surface is covered by Al_2_O_3_ NPs, the lateral size of the final nanocomposite is in micro-scale, rather than in nano-scale. Consequently, the potential toxicity of RGO/Al_2_O_3_ should be diminished due to changes in the material’s dimension. Yet, the potential nanotoxicity of free Al_2_O_3_ NPs is still a matter of concern. For instance, the growth of microalgae *Isochrysis galbana* was significantly inhibited in the presence of nano-Al_2_O_3_ at concentrations over 10 mg L^−1^ (Hu et al., 2018). It was found that Al_2_O_3_ NPs were able to penetrate the cell membrane and aggregate inside within it. This presumably caused the inhibition of microalgae growth, and interestingly, enhancement of cellular fluorescence emission (Hu et al., 2018). What is more, the presence of Al_2_O_3_ NPs enhanced the toxic effects of Pb ions, manifested with increasing bio-uptake of Pb. In this regard, the possible coexistence of nanomaterials and ions should be always taken into consideration in ecotoxicity analysis, since the synergistic effects may occur. On the other hand, the synergic toxicity of Al_2_O_3_ NPs and Cr(VI) ions was not observed in other works (Dalai et al., 2014), since the presence of nanoparticles did not change the bioavailability and uptake of Cr. The solo Al_2_O_3_ NPs were however ecotoxic towards freshwater microalgae, even at the lowest investigated concentration (0.05 μg ml^−1^). The most probable explanation of this issue is their physicochemical properties such as surface area, particle size. This is valid not only in case of GFMs but also for all types of nanomaterials. Their chemical state may change and they can transform while entering different biota, for instance, water or soil environment and therefore, researching the close-to-real conditions may be the key to understanding their ecotoxicity (Peralta-Videa et al., 2011).

## On the RGO/TiO_2_ Nanocomposite System

### Synthesis Approaches

The RGO/TiO_2_ nanocomposites have recently seen an intensive development. It primarily aimed at obtaining photocatalytic systems that could utilize the unique electronic properties of graphene (Liang et al., 2010; Peter et al., 2015). Graphene/TiO_2_ nanocomposites have been obtained using various techniques such as atomic layer deposition (Zhang et al., 2015), liquid phase deposition (Zhang et al., 2011b) as well as a hydrothermal method (Chang et al., 2012), hydrazine reduction, UV-assisted photoreduction, or sol-gel method (Zhang et al., 2010; Guo et al., 2011). Intensive exploration of these techniques has shown that the modification of the graphene surface with TiO_2_ nanoparticles poses many difficulties. For instance, a problem of unsatisfactory bonding between TiO_2_ and graphene surface as well as the problem of TiO_2_ agglomeration was formulated (Zhang et al., 2010; Guo et al., 2011). Other works identified agglomeration of graphene flakes as a consequence of surface modification (Chang et al., 2012), lack of homogenous covering of the RGO surface by TiO_2_ nanoparticles (Liang et al., 2010) and lack of efficient interaction between GO surface groups and TiO_2_ nanoparticles (Williams et al., 2008; Fan et al., 2011). Therefore, the existing methods for surface modification of graphene with TiO_2_ are characterized by numerous disadvantages. Consequently, designing the graphene/TiO_2_ nanocomposites in the form of core@shell structure in which individual (non-agglomerated) 2D RGO flakes (the core) are uniformly covered with TiO_2_ layer (the shell) is a challenge.

**FIGURE 9:**
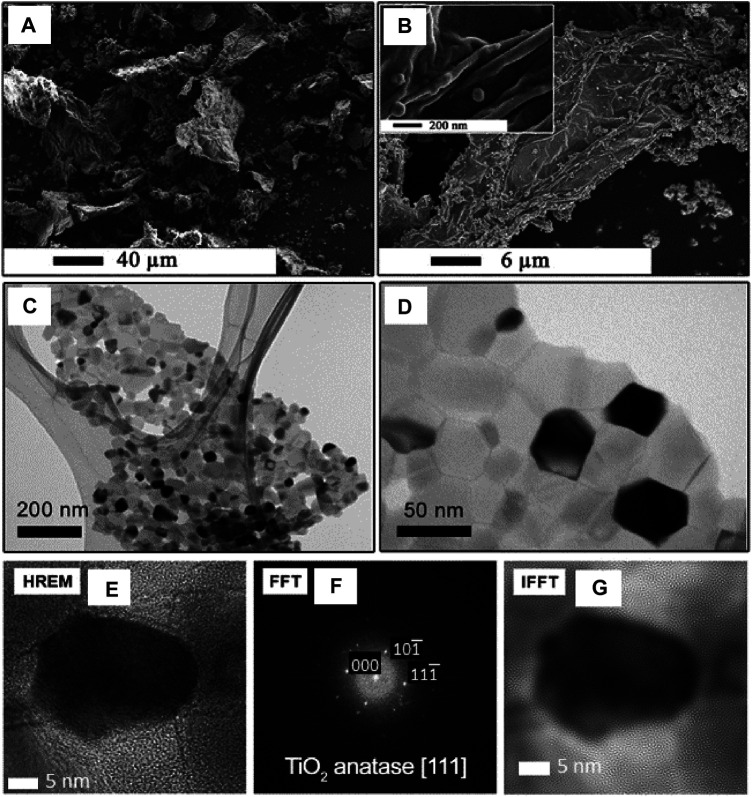
The morphology **(A**,**B)** and structure **(C**–**F)** of the RGO/TiO_2_ (40 wt%) nanocomposite obtained using the simplified sol-gel method analysed with SEM and HREM analysis. The structure of TiO_2_ NPs was analyzed using electron diffraction **(F)** and corresponding IFFT imaging (Reproduced with permission from (Jastrzebska et al., 2016) with minor changes).

Apart from many difficulties in the surface-modification of graphene flakes, the involvement of GO or RGO in synthesis is more promising for RGO/TiO_2_ development. The newly developed *simplified sol-gel method* of synthesis of RGO/TiO_2_ nanocomposite flakes allows for better surface coverage with TiO_2_ NPs and achieving a final product with a more uniform morphology (Jastrzebska et al., 2016). The method involves introducing organic titanium compound into GO flakes dispersion in an organic solvent. Then, the reaction sol is stirred to skip the sol/gel transition. The solvent is then removed and the obtained gel undergoes the air-induced thermal decomposition. The morphology of the RGO/TiO_2_ is shown in Figure 9. The results of the high-resolution TEM and electronic diffraction (Figures 9E–G) confirms the presence of TiO_2_ NPs, mainly in the form of anatase.

### Biocidal Properties

Importantly, the covalent functionalization of GO with TiO_2_ nanoparticles was confirmed also in this case and an *in situ* reduction of GO to RGO (Jastrzebska et al., 2016). A quantitative XPS analysis of changes in chemical composition of the GO surface has shown that the oxygen (in a form of -OH species) was almost completely transferred from the surface of GO to the structure of the titanium tetraisopropoxide (a TiO_2_ precursor). Contrary, the O=C-OH groups were not completely removed from the GO surface. The follow-up study (Jastrzebska et al., 2017d) analyzed the bioactive properties of RGO/TiO_2_ nanocomposites. It was shown that RGO/TiO_2_ stimulated the growth of *S. aureus*, *E. coli,* and *Sarcina* bacteria. Also, no bioactivity was observed in the case of the *Bacillus sp.* strain. Therefore, while the prepared nanomaterial exhibited no biocidal properties, interesting electrostatic properties were noticed in the water environment (Jastrzebska et al., 2016) that were very similar to RGO/Al_2_O_3_ nanocomposites (Jastrzębska et al., 2016a). Development of the core@shell structure has modified the material’s electrostatic properties, which was different from both pure GO and pure TiO_2_ NPs.

### Preparation of Photocatalytic Materials

Recent work introduced the cost-effective and easy method for obtaining good quality RGO/TiO_2_ thin films on fluorine-doped tin oxide (FTO) using a spray pyrolysis technique (SPT) (Alshammari et al., 2020). The homogeneous mixture of TiO_2_ nanopowder and GO dispersion was sprayed onto the FTO glass substrates, which were cleaned before the SPT. After spraying, the oxygen atoms were evaporated and removed from the surface due to the thermal decomposition and annealing process. Contrary, RGO/TiO_2_ nano-flowers were synthesized using a facile, fluorine-free and non-toxic one-pot solvothermal technique (Pugazhenthiran et al., 2020). For this purpose, a dispersion of GO was subjected to ultrasonic treatment and stirring. Subsequently, the TiO_2_ NPs were added into homogeneous suspension and stirred once again. After that, the mixture was autoclaved, centrifuged, washed, and vacuum-annealed. Authors summarized that RGO/TiO_2_ nanocomposite with highly exposed {001} facets was characterized with good crystallization, high dye loading, improved charge transport and enhanced light scattering. The photovoltaic performance was significantly improved as well. The extraordinary photocatalytic dye degradation was shown by RGO/TiO_2_ nanocomposite obtained *via* simple *in-situ* microwave synthesis (Kumar et al., 2015). In this method, GO was synthesized with Hummer’s method and TiO_2_ nanocrystals were homogenized separately in ethanol using ultrasounds. The components were then mixed and stirred. After that, the mixture was exposed to microwave irradiation. Finally, the solvent was evaporated and then, the final product was washed with deionized water and dried. As in the previous cases, the presence of TiO_2_ NPs on RGO surface prevented restacking of RGO layers. Authors also speculated about the role of RGO in the photocatalytic activity of the obtained nanocomposite. They explained that RGO accepts electrons due to its nature, and after being negatively charged, it produces oxygen radicals. Further, because of the reaction with hydroxyl ions, the hydroxyl radicals are being produced. These radicals can degrade organic matter, as they possess large energetic states.

Other techniques for the preparation of RGO/TiO_2_ nanocomposites with homogeneous morphology include the assistance of poly(methyl methacrylate) (PMMA) template using electrostatic assembly (Liu et al., 2018). In this method, PMMA template is redispersed in aqueous solutions and positively charged. Next, carboxylic GO is added dropwise, while the whole mixture is stirred. The next step involves a dropwise introduction of titanium precursor (i.e., tetrabutyl orthotitanate) and stirring. After the process, a nanocomposite is collected, dried in a vacuum and calcined to remove PMMA template. Using this approach, the RGO/TiO_2_ is not agglomerated due to the application of a template. Such powdered RGO/TiO_2_ structure was characterized by the presence of multi-level hierarchical pores and therefore, high specific surface area and large pore volume. It could be beneficial not only in case of biological activity but also hydrogen storage, as hierarchically porous structures allow the rapid diffusion of gases. A similar RGO/TiO_2_ nanocomposite structure was obtained in a work (Olowoyo et al., 2019) using the combined sonothermal-hydrothermal method. It is noted, however, that the procedure was highly complicated. The GO was prepared with modified Hummer’s method and reduced to RGO hydrazine monohydrate in a toluene-based environment. In the next step, RGO was redispersed in water/ethanol solution and homogenized with ultrasounds. After that, few drops of HNO_3_ were added and the mixture was sonicated further, after which it was hydrothermally treated in an autoclave. Next, such obtained product was centrifuged, air-dried, and calcinated. Such composites showed good photocatalytic properties. As authors concluded, it’s really important to find an optimal proportion of TiO_2_ and RGO to improve the photocatalytic properties of the final product. What is more, they observed that RGO is acting as a sensitizer for TiO_2_, which by itself shows little activity under visible light.

Photocatalytic activity of RGO/TiO_2_ nanocomposites has been confirmed in many other studies. In recent work (Guan et al., 2021), the enhanced photocatalytic activity and stability of TiO_2_/graphene oxide composite were obtained for coatings fabricated *via* electrophoretic deposition (EPD). Such composites were able to effectively enhance the photocatalytic activity under visible light, which was associated with forming of TiO_2_ coatings on the surface of GO. What is more, the combination of TiO_2_ and GO was stable, even after ultrasonic cleaning. As shown in (Jin et al., 2021), the photocatalytic activity of TiO_2_ combined with graphene 3D framework may be utilized for efficient adsorption-photocatalytic removal of micro-organic contaminants from water. The synthesis involved rapid freezing-drying followed by high-temperature hydrogen reduction. The 3D RGO/TiO_2_ nanocomposite showed enhanced adsorption-photocatalytic activity for the removal of trace ethenzamide under both UV and vacuum ultraviolet (VUV) irradiation. As authors suggested, the creation of the 3D frame accelerated the transfer of photogenerated electrons, as well as diffusion of micro-organic contaminants. In a study (Yang et al., 2021) it has been shown, that photocatalytic activity of TiO_2_/graphene composite can be also enhanced due to the assembly technique. Authors obtained TiO_2_ core@shell composites covered by graphene sheets *via* a stepwise process. For this purpose, templates were subsequently loaded and heat-treated under an inert atmosphere. Such coating strategy improved the contact area between TiO_2_ and graphene. These nanocomposites were able to separate more carriers generated in the photodegradation process (on both sides of core@shell) and reduce CO_2_ into CO more efficiently. In other work (Tismanar et al., 2021), authors applied the spray pyrolysis deposition (SPD) technique and sol-gel spraying to obtain photoactive TiO_2_/graphene composite thin films. In this regard, factors that may influence the photocatalytic effect were investigated. It has been found that the obtained films were more dye-sensitive in VIS spectra, due to the presence of GO in the composite films. What is more, the efficiency of photodegradation of methylene blue and imidacloprid was influenced mostly by specific surface (roughness) and crystallinity. Furthermore, the washing out effect in an aqueous pollutant environment was not observed, which indicates their stability. Importantly, that such thin films could be also applied to self-cleaning surfaces (Dundar et al., 2020).

## On the RGO/metal Oxide-Me Nanocomposite System

### Preparation of Biocidal Hybrids

The next important step in the development of RGO/metal oxide nanocomposites is the addition of bioactive NPs, which can be composed of various noble metals. The subject literature explores in the majority, the synthesis of graphene-based nanocomposites for use as biocides. The direct decoration method from nanoparticle suspension enabled the modification of graphene surface with nano-Pt, Pd (Xu et al., 2008), Au (Muszynski et al., 2008), Sn (Wang et al., 2009b), and Ag (Chook et al., 2012). Apart from these, only GO/Ag nanocomposite showed biocidal properties. More complicated systems in terms of use for filtration of drinking water include those containing metal oxides. The resulting structure is, obviously, more complex which causes an additional challenge in materials design and optimization. Research in this field was conducted for systems containing TiO_2_ and Ag NPs. In work (Liu et al., 2013), a GO/TiO_2_-Ag nanocomposite demonstrated antimicrobial properties against *E. coli* bacteria. However, its synthesis consisted of mixing GO flakes with separately prepared TiO_2_ fibers and Ag precursor. As a result, Ag NPs were not covalently bonded to the surface of GO flakes.

Two years later, other work (Yang et al., 2015) presented the TiO_2_(P25)/Ag_3_PO_4_/GO composite using electrostatic interactions and the ion-exchange method. In this process, Ag_3_PO_4_ was also mixed with separately-prepared TiO_2_ NPs. The obtained composite efficiently deactivated *E. coli*, *S. aureus*, *Salmonella typhi*, *Pseudomonas aeruginosa*, *Bacillus subtilis* and *Bacillus pumilus*. In another work (Vasilaki et al., 2015), authors obtained the RGO/TiO_2_-Ag nanocomposite using a two-step strategy. In the first step, the photocatalytic nano-TiO_2_ (P25, Degussa) was modified by Ag NPs. Secondly, the GO flakes were modified by TiO_2_-Ag using the hydrothermal method. The authors declared the occurrence of reduction of GO to RGO, however, with no in-depth analysis of the reaction mechanisms. It was also noted that the TiO_2_-Ag NPs did not fully cover the surface of RGO. The abovementioned results show that the most preferred GFM in various synthesis processes is the GO, due to its high surface wettability and corresponding dispersibility. Therefore, the RGO/metal oxide-Me (where: Me = precious metal) nanohybrid system could be an interesting research topic for further studies.

The paper (Jastrzȩbska et al., 2017c) focused on the comparison of bioactive properties of RGO modified by selected NPs of metal oxides such as Al_2_O_3_, TiO_2_, ZnO_2_, SiO_2_ as well as silver NPs (Me = Ag). The RGO/Al_2_O_3_-Ag, RGO/TiO_2_-Ag, RGO/SiO_2_-Ag and RGO/ZnO_2_-Ag nanocomposites were produced using the simplified sol-gel method. The smallest agglomeration was observed for Al_2_O_3_-Ag and TiO_2_-Ag NPs covering the RGO surface (Jastrzȩbska et al., 2017c). In this constellation of nano-compositions, the antibacterial effect was observed for the RGO/Al_2_O_3_-Ag, RGO/TiO_2_-Ag and RGO/SiO_2_-Ag nanocomposites. The strongest biocidal effect against *E. coli*, *S. aureus* and *Bacillus sp.* was observed in the case of the RGO/SiO_2_-Ag nanocomposite. The RGO/TiO_2_-Ag was most efficient against *Sarcina sp*.

**FIGURE 10:**
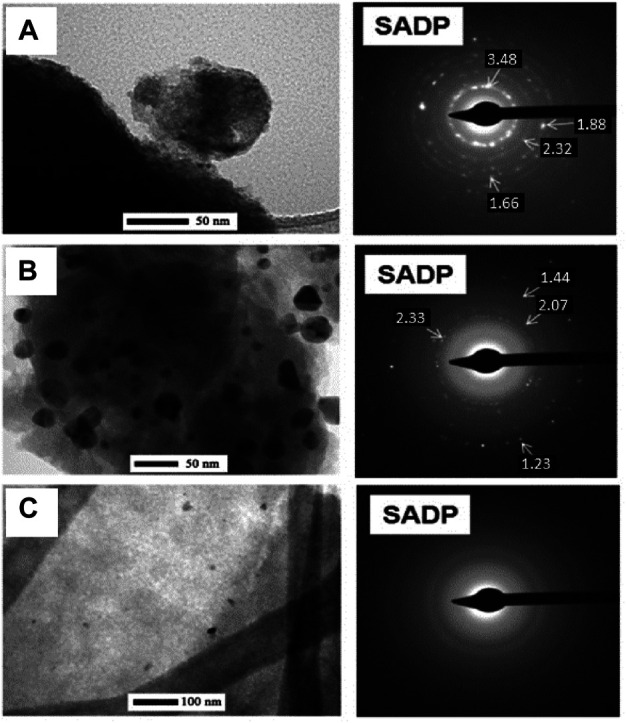
Structural TEM analysis (including electron diffraction (SADP)) with identification of precious metals for nanocomposites such as RGO/TiO_2_ (40 wt%)-Ag (1 wt%) **(A)**, RGO/TiO_2_ (40 wt%)-Au (1 wt%) **(B)**, RGO/TiO_2_ ( 40 wt%)-Pd (1 wt%) **(C)** produced using the simplified sol-gel method (Reproduced with permission from (Jastrzebska et al., 2017d) with minor changes).

In a follow-up study (Jastrzebska et al., 2017d), the works were continued on the RGO/TiO_2_-Me system, where Me was labeled as NPs of precious metals (Me = Ag, Au, Pd). A method of obtaining nanohybrid graphene sorbents from the RGO/TiO_2_-Me system consists of mixing organometallic titanium compound, a precious metal compound, or a mixture of such compounds is added to the graphene flakes or graphene oxide dispersed in an organic solvent. Then, the material is stirred in the presence of dry or wet air, the solvent is removed and the residues after drying undergo air-induced thermal decomposition (Jastrzebska et al., 2017e). Herein, transmission electron microscopy (TEM, *see* Figure 10) revealed the presence of Ag NPs (Figure 10A) and Au (Figure 10B) in TiO_2_ matrix.

**FIGURE 11:**
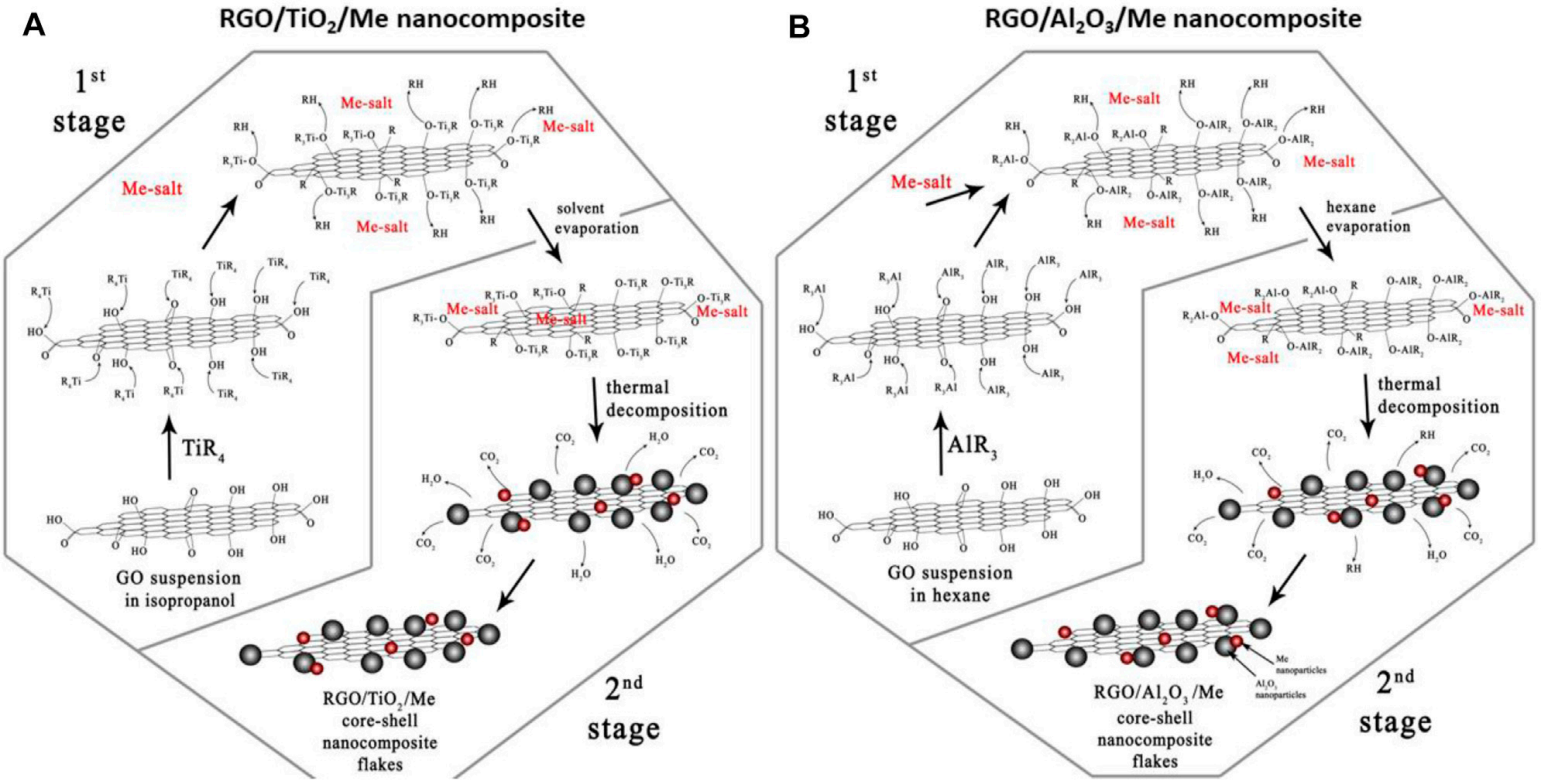
Schematic representation of the mechanism of covalent modification of GO surface with TiO_2_-Me nanoparticles toward nanohybrid RGO/TiO_2_-Me **(A)** and RGO/Al_2_O_3_-Me **(B)** composite systems, using the simplified sol-gel method (Reproduced with permission (Jastrzebska et al., 2017d), (Jastrzębska et al., 2016b), respectively).

The XPS analysis was used for examination of the mechanism of the process of covalent modification of GO surface with TiO_2_-Me NPs, for obtaining hybrid nanocomposite RGO/TiO_2_ (40 wt%)-Me(1 wt%) (Jastrzebska et al., 2017c). The mechanism was schematically presented in Figure 11A. The chemical reactions are taking place mainly between C-OH groups and titanium tetraisopropoxide and involve the co-deposition of silver in the form of silver salts. As a result, -OH groups form the GO surface react with titanium tetraisopropoxide, while no reduction of C=O and O=C-OH residual groups. Then, as a result of thermal decomposition, the removal of part of the organic precursor occurs. Consequently, TiO_2_ NPs and precious metal NPs are simultaneously formed on RGO surface.

Based on the results obtained in (Jastrzebska et al., 2016) and (Jastrzebska et al., 2017e), it was assumed that RGO/Al_2_O_3_ system modified with precious metal NPs is the most promising for further studies. The work (Jastrzębska et al., 2016b) presents a method of RGO/Al_2_O_3_-Me nanocomposite (where Me = Ag, Au, Pd) synthesis. Also in this case, the mechanism of GO reduction to RGO was proposed (*see* Figure 11B). It is noted that a reduction of XPS signals corresponding to C-OH species was observed as well as in previous approaches. Further, the precious metals (Ag, Au, Pd) NPs were co-deposited with Al_2_O_3_ on the surface of RGO.

Other works also involved Cu as the modifying NPs (Sadoun and Fathy, 2019). The solid RGO/Al_2_O_3_-Cu nanocomposite was manufactured using a powder metallurgy technique. The RGO, Al_2_O_3_ and Cu were mixed in ethanol in a planetary ball mill until full homogeneity was achieved and then consolidated. The obtained samples revealed improvement of hardness and wear resistance while increasing the RGO content. What is more, authors also showed a high homogeneity of the obtained nanocomposite, as well as reinforcing grain boundaries due to the presence of RGO. The Al_2_O_3_ coating also improved the morphology, as it allowed to preserve structural voids as well as obtaining the almost spherical shape of nanoparticles. Another important aspect of research is the adhesion between alumina and copper nanoparticles which is much better in comparison to Cu and RGO. Thus, Al_2_O_3_ acts as a bridge and consolidates these into one solid nanocomposite structure, and therefore improves load and transfer capability between Cu and RGO.

### Bioactivity and Biosorption Properties

The RGO/TiO_2_(40 wt%)-Me(1 wt%) nanocomposites were also a subject of bioactivity analysis which demonstrated that the RGO/TiO_2_-Ag nanocomposite showed a growth stimulation of *S. aureus*, *Sarcina* and *E. coli* strains, while the RGO/TiO_2_-Au caused the increased growth of *S. aureus* and *Sarcina*. In case of other samples, no bioactivity was observed. The obtained materials possessed properties stimulating bacterial growth, which showed their potential usefulness as a bacteria culture medium. Results obtained in (Jastrzȩbska et al., 2017c) confirmed the applicability of the RGO/Al_2_O_3_-Ag system for biocidal applications because RGO modified with metal oxides other than Al_2_O_3_ exhibited high agglomeration and/or low biocidal efficiency.

Further analysis of RGO/Al_2_O_3_(40 wt%)-Me(1 wt%) nanocomposites demonstrated their antibacterial activity, but only when Ag nanoparticles were present in the structure. The antibacterial effect was available against the *S. aureus* strain. The presence of other precious metals (especially Pd) caused lower viability for certain bacterial strains (Jastrzębska et al., 2016b). Therefore, the presence of Ag in the material is essential to obtain biocidal properties in the RGO/Al_2_O_3_ nanocomposites.

**FIGURE 12:**
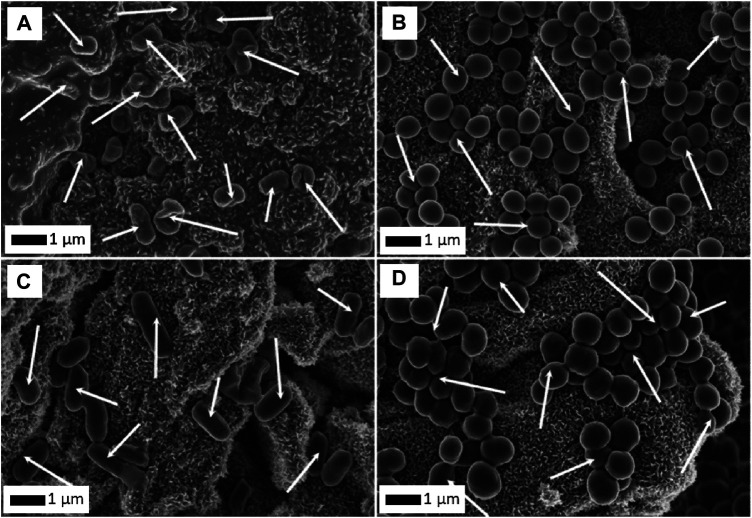
Biosorption of *E. coli* **(A)**, *S. aureus* **(B)**, *Bacillus sp*. **(C)** and *Sarcina* **(D)** cells on the surface of RGO/Al_2_O_3_-Au nanocomposite. Arrows point to bacterial cells (Reproduced with permission from (Jastrzębska et al., 2017e) with minor changes).

The next stage of research involved studies on water filtration systems (Jastrzębska et al., 2017e). The works involved RGO/Al_2_O_3_-Me nanocomposites against *E. coli*, *S. aureus*, *Bacillus sp.*, and *Sarcina sp* bacteria. The system with the addition of Au NPs showed the best bacteria biosorption properties (Figure 12). In our recent work (Jakubczak et al., 2021), we evaluated GO/Al_2_O_3_-Ag hybrid nanocomposite for tap water filtration efficiency. Our system showed high efficiency against both model and waterborne strains microorganisms, stability, reusability as well as self-sterilization ability. Within short periods of contact time, the nanocomposite was able to eliminate up to 100% of the filtered bacteria cells.

**FIGURE 13:**
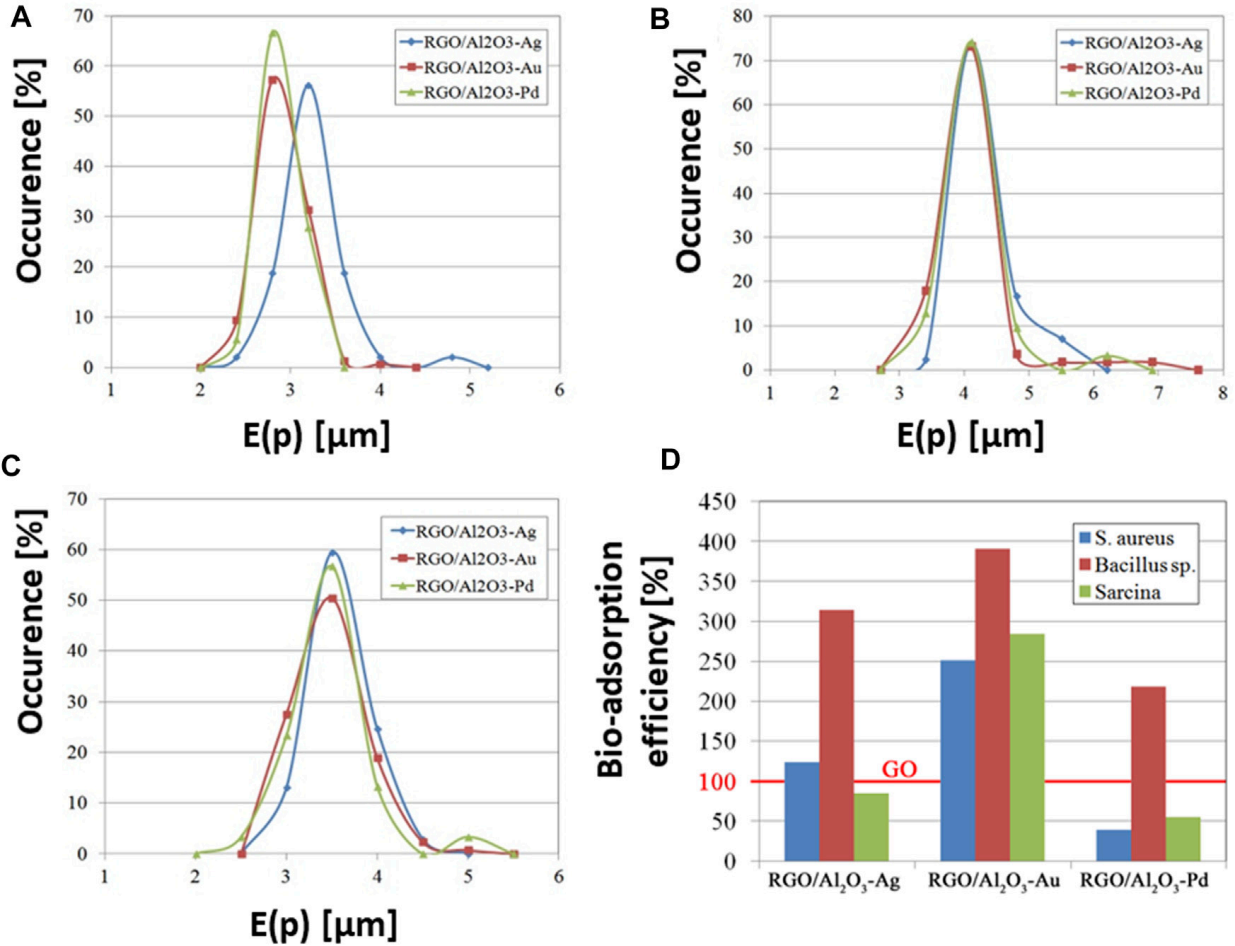
Analysis of changes in the shape of bacteria cells absorbed on the surface of the RGO/Al_2_O_3_-Me nanocomposites, presented as distributions of E*(p)* values for *S. aureus* **(A)**, *Bacillus sp.* **(B)**, and *Sarcina* **(C)** strains (Reproduced with permission from (Jastrzębska et al., 2017e)). Bacteria adsorption efficiency tested for RGO/Al_2_O_3_-Me nanocomposites in comparison to the reference GO flakes **(D)**, for which the benchmark of the 100% efficiency is assumed (Reproduced with permission from (Jastrzębska et al., 2017e) with minor changes).

The quantitative analysis of morphology for bacterial cells adsorbed on the surface of the RGO/Al_2_O_3_-Me nanocomposites was carried out as part of the recent work (Jastrzębska et al., 2017e). The quantitative analysis was carried out for the *S. aureus, Bacillus sp.* and *Sarcina* strains using the method applied in the microstructural analysis of nanomaterials–the stereological analysis described in (Jastrzębska et al., 2016b) and (Jastrzebska et al., 2016). The stereological approach was applied for the first time to carry out a quantitative analysis of the morphology of bacterial cells (Jastrzębska et al., 2017e). The *E(p)* parameter was used to describe the true shape of bacterial cells. It was chosen from a broad range of various stereological parameters employed in graphical analysis of changes in the morphology of individual bacteria cells, resulting from the interaction with the RGO/Al_2_O_3_-Me nanocomposite surface. The E*(p)* distributions designated for the *S. aureus* cells (Figure 13A) adsorbed on the surface of the RGO/Al_2_O_3_-Au and the RGO/Al_2_O_3_-Pd were comparable, which suggests a lack of changes in cell morphology. The analysis of cells after adsorption on the surface of the RGO/Al_2_O_3_-Ag nanocomposite showed shifting of E(*p*) towards higher values. Such a result suggests an increase in cell shapes directly associated with morphological changes (more irregular surface of the cell wall or cells’ inflation/shrinkage). The observed effect was linked to the presence of biocidal Ag NPs in the surface structure of the RGO/Al_2_O_3_-Ag nanocomposite. Using the method of quantitative analysis of cell morphology, it was possible to detect even small changes in E(*p*) values for *Bacillus sp.* (Figure 13B) and *Sarcina* (Figure 13C) cells adsorbed on the surface of the RGO/Al_2_O_3_-Ag (Jastrzębska et al., 2017e).

Another important approach is the analysis of biosorption efficiency for the RGO/Al_2_O_3_-Me nanocomposites (Figure 13D). Such quantitative analysis is based on a determination of the number of bacteria adsorbed per unit of nanocomposite area. The reference material was GO flakes, for which the 100% adsorption efficiency was assumed as a benchmark (Xu et al., 2008). Obtained results indicate that the RGO/Al_2_O_3_-Au nanocomposite showed the best bio-adsorption efficiency for *S. aureus* (6.1 cells/10 µm^2^), *Bacillus sp.* (1.9 cells/10 µm^2^), and *Sarcina* (13.1 cells/10 µm^2^), while the RGO/Al_2_O_3_-Ag exhibited a medium level of biosorption efficiency. For the case of RGO/Al_2_O_3_-Pd nanocomposite, the lowest biosorption effect was observed.

### Preparation of Electroactive Hybrids

In other work (Sangjan et al., 2019), RGO/TiO_2_-Ga and RGO/TiO_2_-Gd nanocomposites were successfully obtained using a simple procedure. Starting reagents were stirred together and then separated *via* centrifugation. Next, samples were washed using deionized water and dried in the air. In further research, obtained materials were characterized and investigated in terms of photocatalytic dye degradation. It was found that the introduction of metals allows for more effective degradation of the blue dye. It is noted that after reaching a certain percent addition of both Ga and Gd, there was no further improvement in efficiency because a key factor here is an optimization of metal content. As authors concluded, the addition of metal into RGO/TiO_2_ photocatalyst increases sub energy band gap of TiO_2_ and decreases the photoresponse energy. However, at the high concentration, material agglomerates with TiO_2_. This reduces the number of active sites and decreases photoreaction efficiency. The enhanced photoelectrocatalytic activity of the TiO_2_ was also obtained in work (Dargahi et al., 2018). For that, authors doped Mo with titanium using the mechanical alloying technique, which was then hybridized with RGO. Mo-doped TiO_2_ and GO were mixed and homogenized using ultrasounds. Next, water was removed from the sample, replaced with absolute ethanol and once again sonicated. In the end, the suspension was exposed to UV light (350 nm) and dried afterward. The fabricated nanocomposite was characterized by a significant enhancement in the photogenerated current (7.8 times higher compared to the bare TiO_2_), which corresponded to the reduction of the band-gap energy of the TiO_2_, as well as the efficient separation and prolonged recombination time of the charge carriers.

As shown in another study (Ahmadi et al., 2018), the photoelectric properties can be improved by doping RGO/TiO_2_ nanocomposite with Ce, instead of Mo. Authors doped TiO_2_ with Ce using the sol-gel method, in which precursor reagents were mixed separately with absolute ethanol. Next, both suspensions were combined, stirred, and pH was adjusted to start sol-gel transformation. Thus obtained gel was then dried and calcinated, which ended the procedure. Further, RGO/TiO_2_-Ce nanocomposite was synthesized using the hydrothermal method (Ahmadi et al., 2018). Firstly, GO was dispersed in absolute ethanol and deionized water, after which the suspension was homogenized with ultrasounds. Next, Ce-doped TiO_2_ NPs were added and the whole suspension was stirred until full homogenization. The last step involved placing the suspension into a teflon-sealed autoclave and heating, after which it was filtered and dried to obtain the final sample. As was shown, the RGO/TiO_2_-Ce photoelectric properties were rather different in comparison to its bulk counterpart. Combining reduced graphene oxide, titania and Ce resulted in a shift of absorption spectra toward visible light, decrease in photoluminescence emission (70% lower than reference TiO_2_), high electronic conductivity, reduction of the charge carrier recombination rate and increase of the electrical conductivity.

The influence of different geometries of Ag NPs in improving the photocatalytic efficiency of RGO/TiO_2_-Ag nanocomposite was also investigated (Chen et al., 2020b). Authors applied Ag nanospheres, nanodecahedrons and nanoprisms, which have been prepared separately and then, incorporated during the nanocomposite synthesis. As synthesized Ag NPs dispersions were mixed with TiO_2_ and then, the RGO was added. The mixture was then stirred, homogenized, dried in an oven, and ball-milled until the homogenous powder was formed. Further analysis revealed that the introduction of all Ag nanostructures allowed for enhancing the absorbance of co-catalyst in both UV and visible light ranges. What is more, nanocomposite with Ag nanodecahedrons showed a broader absorption in the long-wavelength region (in comparison with Ag nanospheres). This was explained by the presence of localized surface plasmon resonance effect for Ag nanodecahedrons. Due to the multiple-resonance property of Ag nanoprisms, the resulting RGO/TiO_2_-Ag nanocomposite was characterized by extinction enhancement that was stretched all over the visible light spectrum. The absorption was enhanced not only by the addition of Ag nanoparticles but also the introduction of RGO, which strongly affected the redshift in the absorption edge. The importance of not only improving materials properties but also synthesis using environmentally friendly techniques (green synthesis) was recognized in work (Zhang et al., 2017). Authors obtained RGO/TiO_2_-Pd nanocomposite by a simple two-step process using hydrothermal synthesis and reduction. For that, the GO, synthesized with modified Hummers’ method, was dispersed in deionized water and homogenized using ultrasounds. In the next step, titania precursor was added, and the mixture was stirred under ultrasounds, after which it was transferred into an autoclave. Thus obtained samples were washed with water and dried under vacuum. Such nanocomposites were then doped with Pd. After redispersing and ultrasound homogenization, the Pd precursor was added and the whole was mixed. Lastly, the KBH_4_ solution was added dropwise to reduce Pd^2+^ under constant stirring of the mixture. Noticed improvement of electrochemical performance was assigned to the synergic effect of TiO_2_-Pd, which was supported by the large specific surface area and superior conductivity of RGO.

## Conclusion and Outlook

The development of nanohybrid systems enriched with graphene family nanomaterials (GFMs) hold great promise for innovations in the area of bioactive materials as well as various water treatment technologies. The GFM/metal oxide-bioactive metal nanohybrids can be obtained using the covalent surface modification of GFMs with nanoparticles. To be useful in the filtration of drinking water and its decontamination, they should exhibit both biocidal and biosorption properties to bacterial cells and/or catalytical features. Recent developments in the field are, unfortunately, scarce. However, they allow for important assumptions on the expected properties. The modification of GFMs with nanoparticles needs a careful examination of material morphology, structure, and functional properties. In this regard, understanding the general surface activity, bioactivity and the associated sorption phenomena are of high interest. These are also a must to enabling moving forward with nanotechnology development and further upscaling. The most substantial steps in this area include designing the structure and chemical composition of hybrid systems, developing innovative methods for covalent modification, and optimization of synthesis parameters. In this regard, confirmation of the existence of covalent bonds between the surface of the GO and nanoparticles plays a vital role together with a comprehensive description of morphology, structure, and physicochemical properties of the obtained bioactive and biosorption nanocomposite systems.

Within the most recent research in the field of bioactive GFM-based hybrids, it was demonstrated that the developed RGO/Al_2_O_3_-Me nanocomposites are characterized by the best biosorption properties for *Bacillus sp.* and *E. coli* cells, which confirmed their further applicability in the filtration of drinking water. The RGO/Al_2_O_3_-Ag nanocomposite was recognized as the most effective material as it possessed both biosorption and biocidal properties. This last characteristic is corresponding to nanosilver content. The combination of these two parameters enabled better attraction of bacteria cells to the surface of the RGO/Al_2_O_3_-Ag nanocomposite and their subsequent complete deactivation. The already made effort is a great step forward development of complex GFM-based structures. On the other hand, more research is needed to further confirm the applicability of these innovative materials in larger scale, up to the industrial practice.
